# Layered calcium phenylphosphonate: a hybrid material for a new generation of nanofillers

**DOI:** 10.3762/bjnano.9.269

**Published:** 2018-11-20

**Authors:** Kateřina Kopecká, Ludvík Beneš, Klára Melánová, Vítězslav Zima, Petr Knotek, Kateřina Zetková

**Affiliations:** 1 Department of General and Inorganic Chemistry, Faculty of Chemical Technology, University of Pardubice, Studentská 573, 532 10 Pardubice, Czech Republic; 2 Joint Laboratory of Solid State Chemistry, Faculty of Chemical Technology, University of Pardubice, Studentská 84, 532 10 Pardubice, Czech Republic; 3 SYNPO, akciová společnost, S. K. Neumanna 1316, 532 07 Pardubice, Czech Republic; 4 Institute of Macromolecular Chemistry of the Czech Academy of Sciences, Heyrovského nám. 2, 162 06 Prague 6, Czech Republic

**Keywords:** exfoliation, layered phenylphosphonate, nanomaterial, nanofiller, polymer filler

## Abstract

The use of nanosheets of layered calcium phenylphosphonate as a filler in a polymeric matrix was investigated. Layered calcium phenylphosphonate (CaPhP), with chemical formula CaC_6_H_5_PO_3_∙2H_2_O, is a hybrid organic–inorganic material that exhibits a hydrophobic character due to the presence of phenyl groups on the surface of the layers. In this paper, various CaPhP synthesis methods were studied with the aim of obtaining a product most suitable for its subsequent exfoliation. The liquid-based approach was used for the exfoliation. It was found that the most promising technique for the exfoliation of CaPhP in an amount sufficient for incorporation into polymers involved using propan-2-ol with a strong shear force generated in a high-shear disperser. The filler was tested both in its unexfoliated and exfoliated forms for the preparation of polymer composites, for which a low molecular weight epoxy resin based on bisphenol A was used as a polymer matrix. The prepared samples were characterized by powder X-ray diffraction, atomic force microscopy, optical and scanning electron microscopy, and dynamic mechanical analysis. Flammability and gas permeation tests were also performed. The addition of the nanofiller was found to influence the composite properties – the exfoliated particles were found to have a higher impact on the properties of the prepared composites than the unexfoliated particles of the same loading

## Introduction

The idea to combine materials with different properties to create a composite that benefits from a synergistic effect and to gain better and novel materials by this way is a very old concept. The reinforcement of a polymer matrix with inorganic fillers with the aim to improve their stiffness, melt behavior, mechanical characteristic, durability and other properties of polymer products is a well-known process and has been studied for decades. Platelets of clay minerals are suitable and widespread fillers. In addition, some clays can be exfoliated thanks to their layered structure and thus fillers formed from nanosheets can be obtained [1–2]. Convenient interactions among functional groups of the polymer chain and the surface groups of the filler are necessary to retain the nanoscale character of the filler and to protect it from the formation of agglomerates in order to achieve a homogenous distribution within the volume of the polymer matrix. This is a drawback for natural clays as they are usually hydrophilic, thus their application is more suitable for water-based systems. This limitation can be overcome by intercalation of organic molecules into the structure or by a surface modification [3–4] by grafting organophilic functional groups onto the clay surface, leading to the synthesis of hybrid organic–inorganic materials [5].

Layered metal organophosphonates are a class of materials which exhibit a hybrid character by their nature. They are generally defined as salts of phosphonic acids with the general formula RPO_3_H_2_ (R = alkyl or aryl group) with metals. They benefit from a well-defined inorganic structure in combination with organic moieties which can be functionalized and modified to obtain desired properties. Many different types of layered metal organophosphonates have been prepared. Well-known and well-studied are the organophosphonates of zirconium because of their good stability; however, it is also possible to prepare layered structures with divalent metals such as calcium, strontium or barium [6–9]. Although the properties of these compounds differ with the metal and organic group incorporated in their structure, the main characteristic remains the same: a strong in-plane bonding in combination with weak van der Waals interactions between the planes. This arrangement enables their use as a host material in intercalation chemistry and as a precursor for the preparation of nanosheets by exfoliation.

Exfoliation is a process whereby thin sheets of material are completely separated from the bulk. This happens when cohesive forces between the adjacent planes, which are usually caused by van der Waals interactions, are overcome. Mechanical or chemical action can be involved. Various exfoliation methods have been studied mainly for the exfoliation of graphite to produce graphene [10], but the main ideas and approaches are also applicable for other types of layered compounds. In this work a so-called liquid-based-exfoliation process was used [11–15], which is considered to be convenient for the production of larger quantities of material for further application. Briefly, particles are dispersed in a suitable liquid and then exfoliated by a force action; this can be done with or without adding further chemicals to weaken cohesive forces. The main advantage is that a ready-to-use dispersion of nanoplatelets is obtained, so the step of dispersing dry nanoparticles in a polymer matrix is avoided, which is usually challenging, and thus simplifies the preparation of the polymer composites.

**Figure 1:**
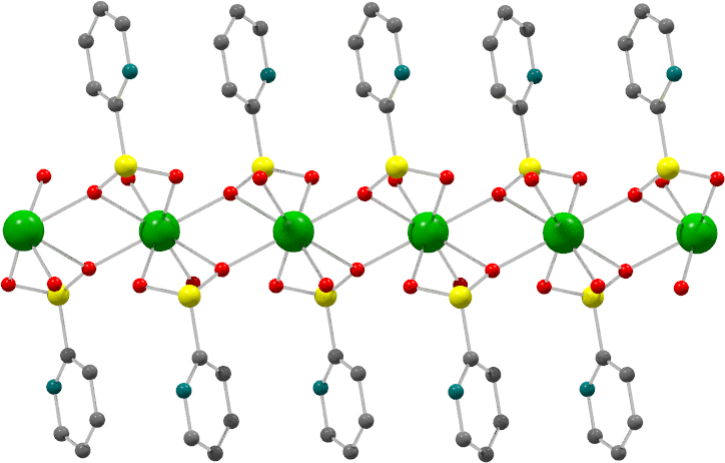
Model structure of the CaPhP layer. Calcium atoms are in green, phosphorus atoms in yellow and red and gray spheres correspond to oxygen and hydrogen, respectively.

In this work, layered calcium phenylphosphonate dihydrate with formula CaC_6_H_5_PO_3_∙2H_2_O (CaPhP) was used both in exfoliated and unexfoliated forms to prepare a polymer composite with the intention to move towards applied science and find possible applications. The CaPhP layer can be imagined as a sheet consisting of three plies. The central ply comprises metal atoms coordinated by oxygen atoms of the phosphonate groups. The outer plies are formed by benzene rings connected to the central ply through phosphorus atoms of the phosphonate groups. The model of the CaPhP layer is depicted in Figure 1. Thanks to its structure, the surface of the CaPhP layer is formed by rather hydrophobic phenyl groups, which makes them a suitable material for incorporation into the hydrophobic polymer matrices. An epoxy resin was chosen as a polymer matrix because epoxy resins are widespread and useful in various industrial applications ranging from coatings, to adhesives, to the preparation of composites in automotive or aerospace industry [16–17]. The experiments were carried out with an unmodified low molecular weight epoxy resin based on bisphenol A, which is one of the basic ones.

## Results and Discussion

### Filler synthesis

The layered structure of calcium phenylphosphonate is formed at room temperature by a co-precipitation of starting compounds – phenylphosphonic acid and a soluble source of calcium (e.g., CaCl_2_, as in our case) in a 1:1 molar ratio at pH > 9, as reported by Svoboda et al. [18] and described in the Experimental section. The previous work was focused on the material itself, characterization of its chemical composition, behavior under different pH and its intercalation chemistry. In this work, this material was studied from the point of its application as a filler in a polymer matrix, which implies that possible ways for large-scale synthesis of this material were investigated. Therefore, the experimental procedure and its conditions were optimized with the aim to obtain a pure product on a large scale.

**Figure 2:**
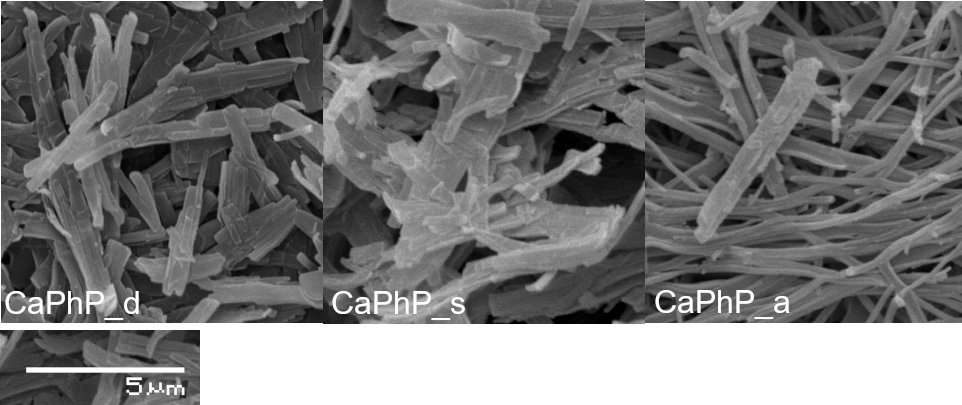
SEM pictures of calcium phenylphosphonate particles prepared by addition of chloride solution using the “drop by drop” (CaPhP_d) and “in several portions” methods (CaPhP_s) and “all at once” (CaPhP_a).

**Figure 3:**
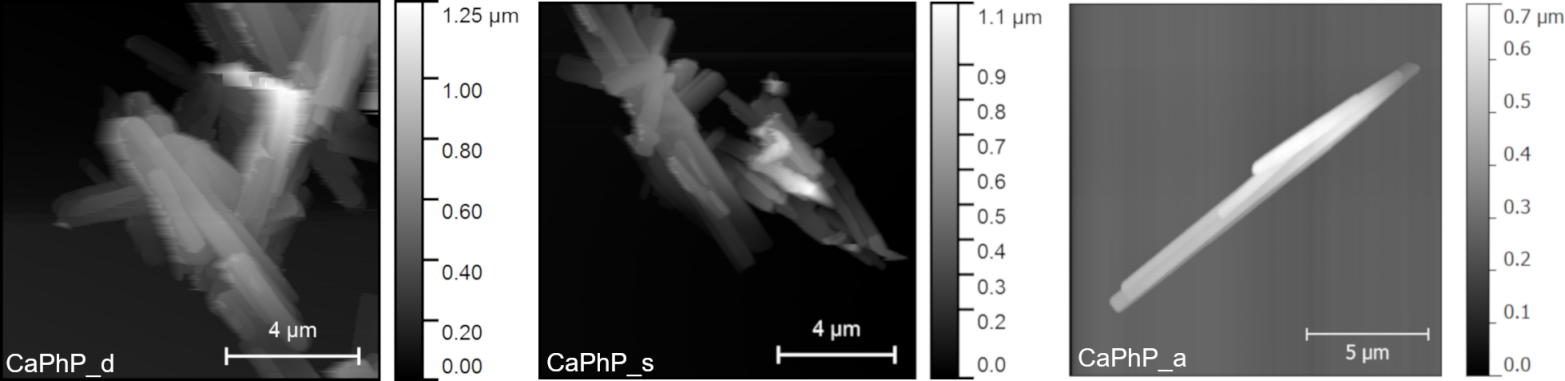
AFM images illustrating the topology of CaPhP particles prepared by addition of calcium chloride solution: “drop by drop” (CaPhP_d), “in several portions” (CaPhP_s) and “all at once” (CaPhP_a).

There are two points which need to be highlighted: First, the role of pH is crucial. To obtain the desired product, it is necessary to maintain a pH at around 9 all the times during the reaction process and in the whole reaction volume. If this condition is not fulfilled, an intermediate with formula Ca_3_(C_6_H_5_PO_3_)_2_(C_6_H_5_PO_3_)_2_∙4H_2_O [18], which is formed near neutral pH, cannot be transformed into the desired CaPhP but remains present as an impurity in the final product. This intermediate can be identified as another layered phase in the XRD pattern with basal spacing around 15.2 Å. What is more important from the technological point of view, the reaction mixture tends to be denser, which causes problems with homogenous stirring. Over the course of the reaction, the pH decreases to about 8 during the first 15 minutes and then remains the same. Thus, it is recommended to start at pH 10 when working with higher volumes, even if it means a few more rounds of washing to remove ammonium in the final step. Second, it is appropriate and advantageous to add the whole volume of calcium chloride solution at once into the reaction mixture. Two methods, the "drop by drop" addition and addition in several portions, were tested in an effort to achieve larger but thinner particles as it is expected that the edges of already formed lamellas could act as nucleation centers; however, these methods were not successful. Although the shape of the particles differs minimally as they are rod-shaped in each case, the thickness of the individual lamellas differs significantly with the selected procedure, as was confirmed by the atomic force microscopy (AFM) analysis. The samples prepared by the “drop by drop” and “several portions” methods contained thinner particles in comparison those where the portion was all added at once. Nevertheless, as it can be seen in scanning electron microscopy (SEM) images (Figure 2) and as was also verified by AFM (Figure 3), in the first two mentioned cases, the lamellas tend to grow together into aggregates. A possible explanation is that the successive addition of the reactant causes preferential growth of new lamellas on the existing lamellas rather than being formed separately. Such formations are not so useful for application as a polymer filler where individual particles are more desirable. Thus, it was preferable to prepare thinner lamellas by exfoliation and not by varying the rate of the reactant addition.

### Preparation of the nanofiller – exfoliation

**Figure 4:**
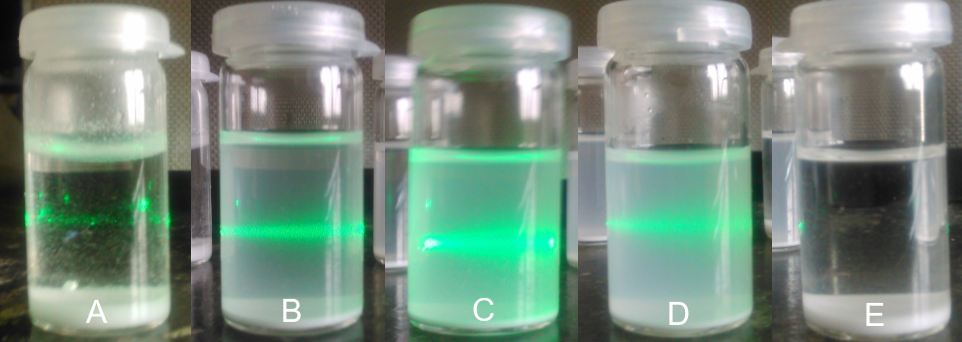
CaPhP nanoparticles in various solvents 24 hours after ultrasound treatment (A – distilled water, B – propan-2-ol, C – ethanol, D – butanol, E – acetone). The green line is Tyndall scattering by a green laser.

Exfoliation is a top-down approach that can be used to obtain nanostructures [12–13]. As was mentioned in the Introduction, calcium phenylphosphonate, by its layered nature, should be a good candidate for the delamination. As previously described by several research groups, solvent compatibility with exfoliated material is one of the key parameters for successful delamination [13–14]. If the solvent–material interaction is not favorable, delamination could likely be observed within a short time; however, the dispersion of the resulting nanosheets will not be stable enough for further application. There is the possibility to extend the duration of the nanosheet dispersion by adding surface-active agents but choosing an appropriate one is also not trivial. Based on this consideration, this work was focused on a selection of suitable solvents which produce stable dispersions without the addition of other chemicals. As described in the Experimental section, an ultrasound treatment was applied to a combination of calcium phenylphosphonate with different solvents and then the dispersion stability was observed up to 24 hours after the ultrasound treatment. The presence of small particles in the dispersion was confirmed by Tyndall scattering of the green laser beam (see Figure 4).

It is possible to conclude that this material is not compatible with water or acetone. Alcohols were shown to be the best solvents for this type of exfoliation, namely propan-2-ol and *n*-butanol. In fact, the stability of the dispersion in *n*-butanol was slightly better than in propan-2-ol; however, *n*-butanol is not so convenient for application in the composites due to its higher boiling point. Also, ethanol seems to be a promising solvent, however, ethanol is polar and thus less compatible with hydrophobic substances. Therefore, propan-2-ol was chosen, which is more appropriate for treatment with hydrophobic compounds and, in comparison to pure ethanol, less expensive.

The exfoliation of CaPhP in propan-2-ol was studied using various force actions starting with sonication, in addition to the combination with mild shear force, which is produced by pushing the particle dispersion through an injection needle by a peristaltic pump. Additionally, a strong shear force created by a high-shear disperser, where the velocity of dispersion was 5 m/s, was also applied. It was found out that the most suitable method for the exfoliation of calcium phenylphosphonate was the strong shear force applied by using the high-shear disperser.

**Figure 5:**
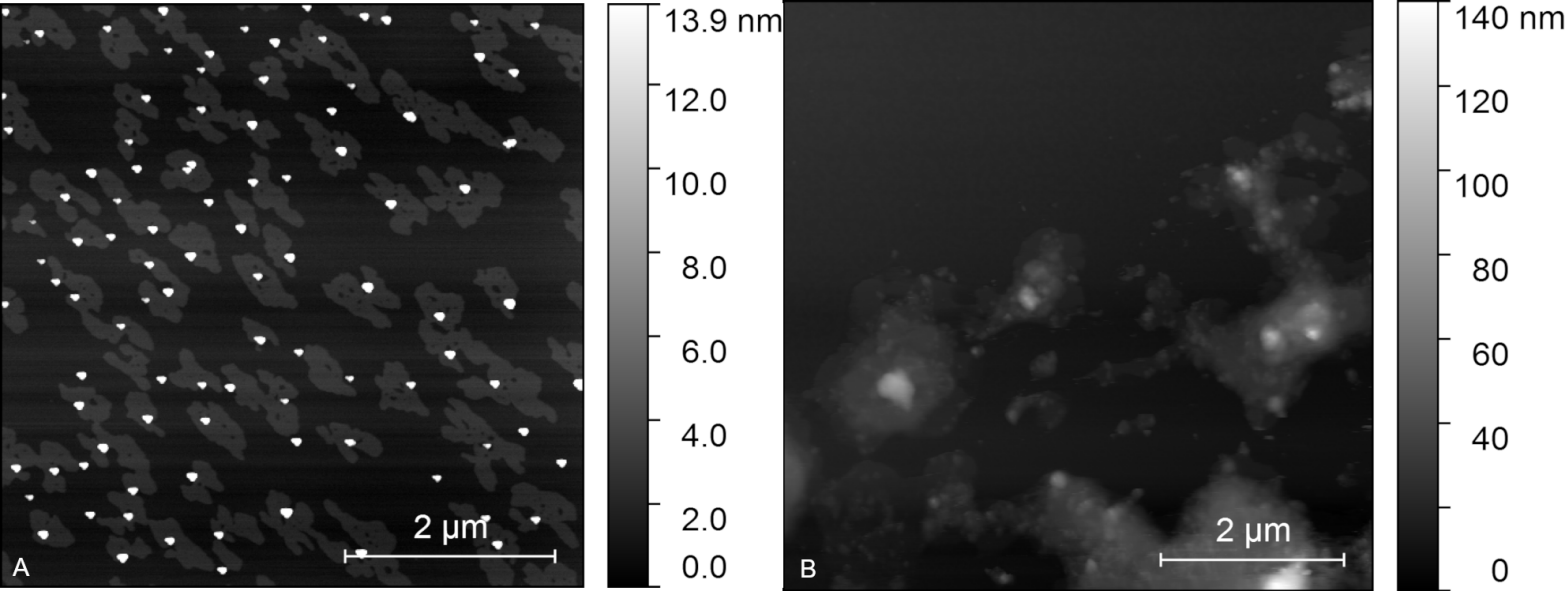
AFM pictures of exfoliated CaPhP: A) Particles successfully exfoliated into monolamellar sheets. B) Material destroyed by too long and too strong mixing.

These dispersion methods are primarily used for breaking down agglomerates; however, in the case of such layered compounds, as this material is, the force is strong enough to not only disperse but also to delaminate the particles. As can be seen in Figure 5A, one can get nanosheets that are 1.4 nm thin but with lateral dimensions of hundreds of nanometers. What is important is that there are also industrial-scale dispersion apparatus available, so this approach is not limited only to laboratory use.

**Figure 6:**
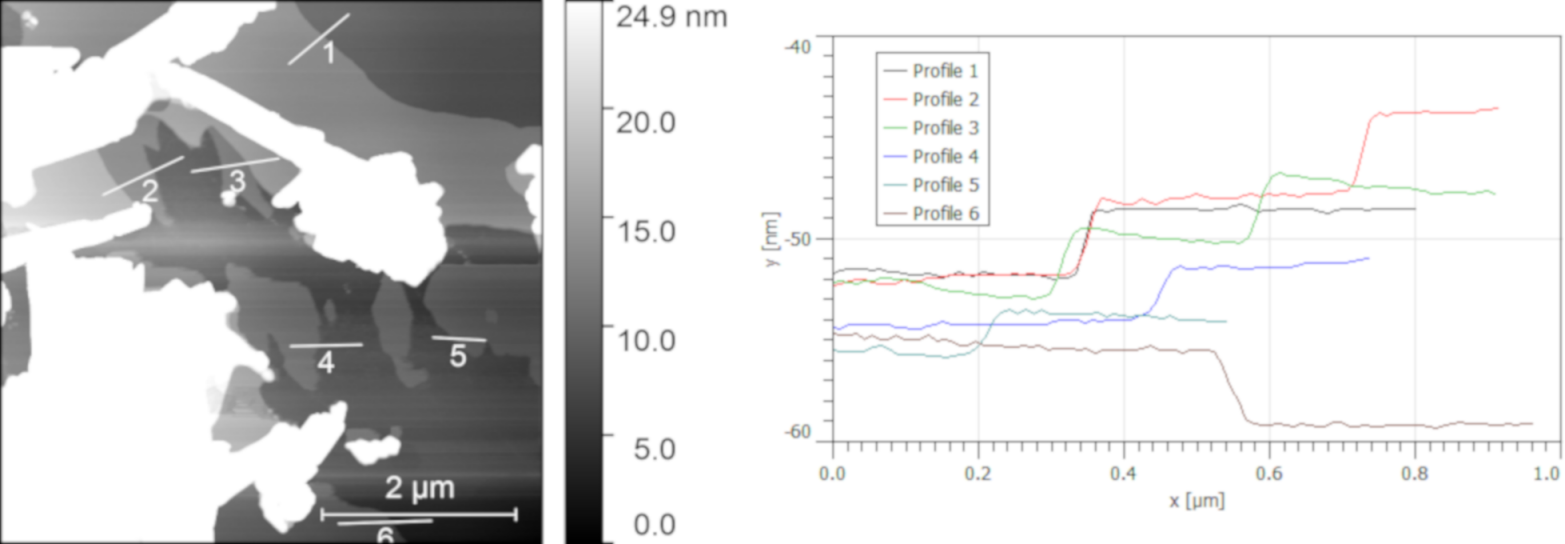
AFM topology scan of exfoliated CaPhP: monolamellar sheets (light gray areas), incompletely exfoliated particles (white areas) and line profiles (right) corresponding to the marked regions in the image to the left.

However, there are also limits that need to be mentioned. From our experience, it is not possible (at least for CaPhP) to fully exfoliate all particles. The obtained dispersion is always a mixture of monolamellar and multilamellar entities (Figure 6).

It is possible to influence the exfoliation yield by the time applied and by the speed of the rotor but too long and too high speed (thus too strong force) lead to the complete destruction of the particles. A result of too long mixing is documented in Figure 5B. There are no visible particles with a lamellar structure or at least with a regular shape, therefore this product is not convenient for application as a nanofiller. The separation of fully exfoliated particles is possible by centrifugation but this also leads to a loss of the material, thus, it is more suitable for a small-scale sample preparation and not so convenient for large-scale production. Nevertheless, even exfoliation from hundreds of layers to particles producing only 20–30 layers can significantly influence the behavior of the produced material when it is used as a polymer filler.

### CaPhP as a filler in epoxy resin

#### Dispersion quality

**Figure 7:**
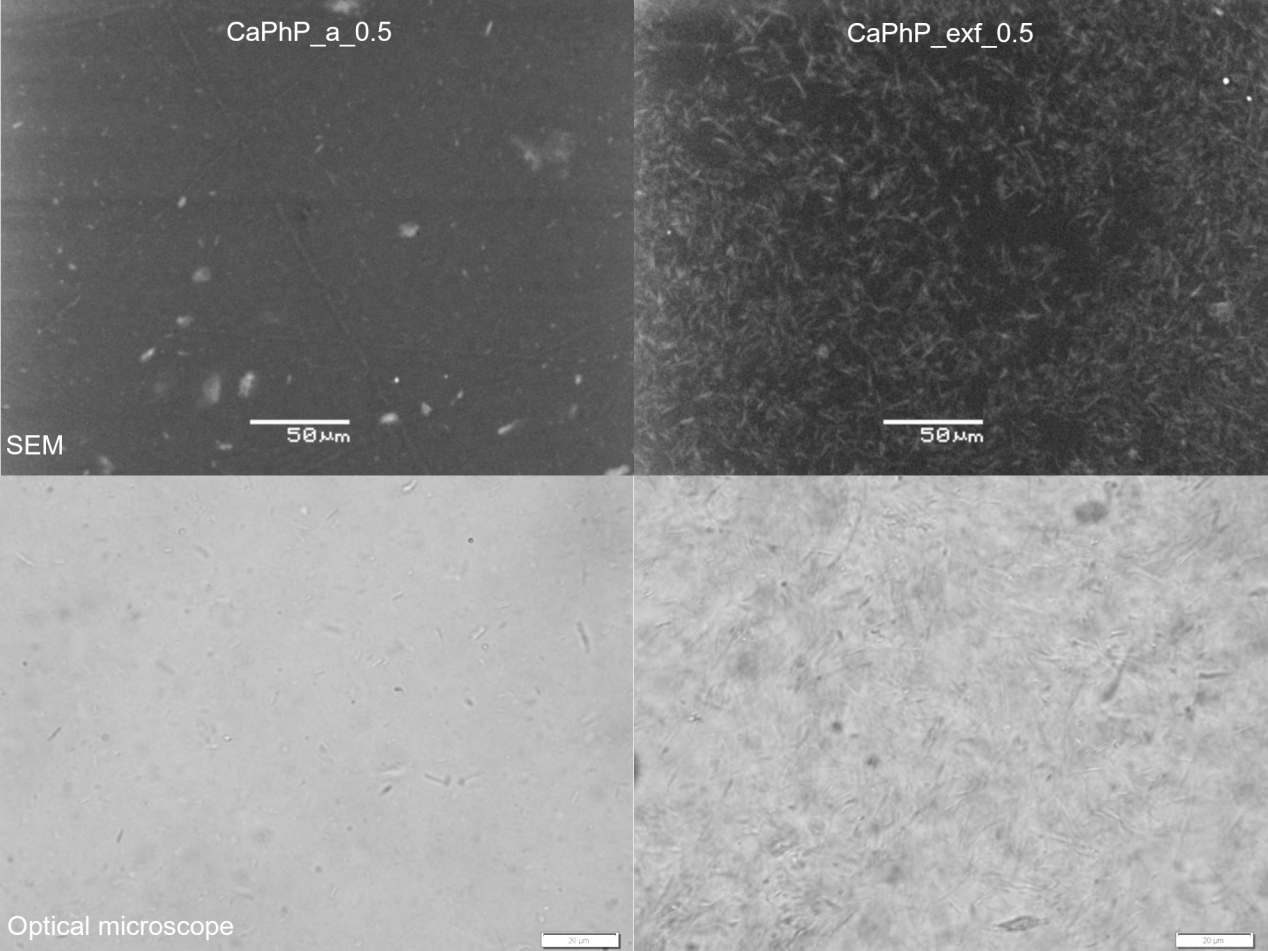
Pictures of the composite-free films with 0.5 wt % loading of the filler obtained from the scanning electron microscope and from the optical microscope. Scale bars in the optical microscope pictures are 20 µm.

To benefit from the filler properties, it is necessary to disperse the material well in the polymer matrix. The pristine calcium phenylphosphonate is able to form a stable and fine dispersion without visible agglomerates in the used CHS-EPOX 520 epoxy resin by a three roll milling, which is a standard polymer processing procedure in which high shear forces between rotating cylinders are used to break down the agglomerates. This dispersion is stable for months without sedimentation and visible changes. In the case of exfoliated lamellas, a nice dispersion without visible agglomerates was obtained even by mixing it with an ordinary dispersing disc. The particle distribution within the polymer matrix is homogenous and no large agglomerates are present. This is illustrated in pictures of the composite-free films obtained from the optical microscope and scanning electron microscope using a back-scattered electron detector to visualize the chemical contrast (Figure 7). This figure also reveals the difference between the unexfoliated filler (CaPhP_a) and the exfoliated nanoplatelets (CaPhP_exf). In both cases, the particles are homogenously dispersed in the whole volume of the matrix. However, in the case of the exfoliated filler (CaPhP_exf_0.5), significantly more individual particles can be detected than in the case of the unexfoliated filler (CaPhP_a_0.5).

This supports the idea that, in the case of the nanofiller, significantly less material is needed to produce the desired volume of filler. Of course, the nanoscale dimensions of the filler do not guarantee a proper particle distribution within the polymer matrix, and it is therefore also very important that the chemical nature of the particles and their compatibility with the polymer chains are also considered.

#### Interaction with the polymer blend components

**Figure 8:**
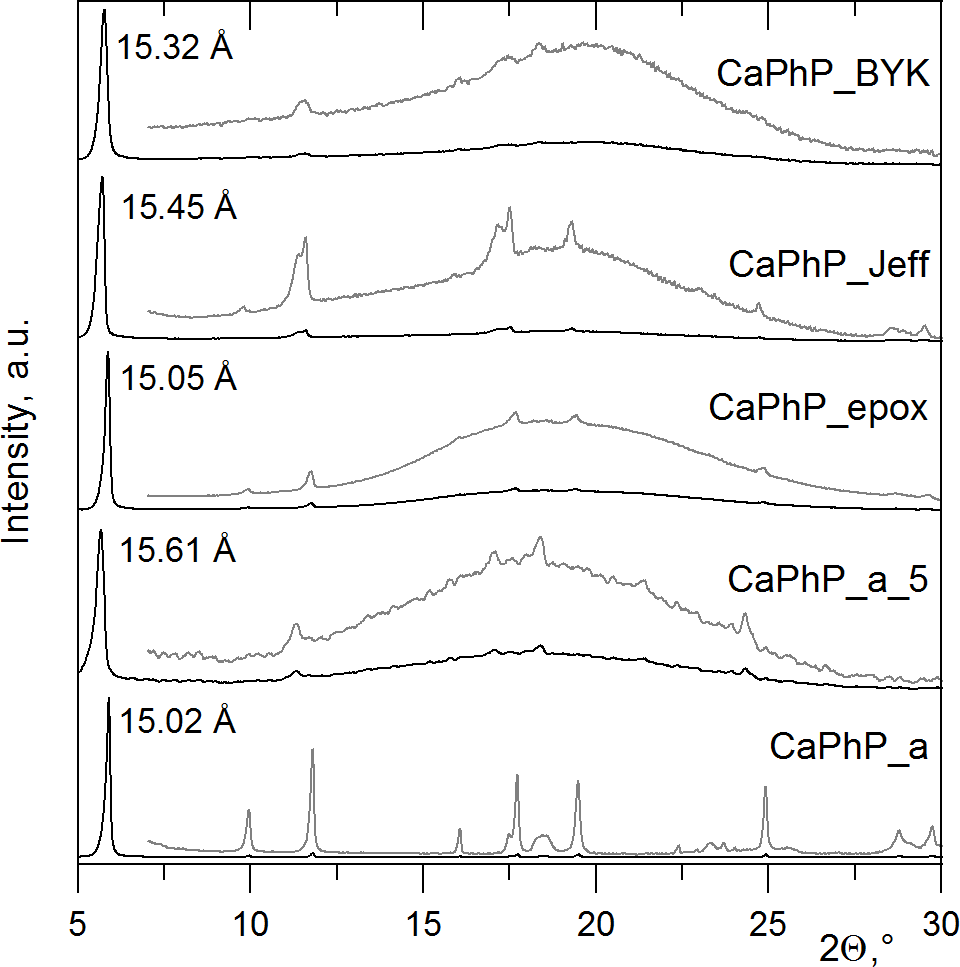
XRD patterns of original calcium phenylphosphonate (CaPhP_a), free film with 5 wt % of CaPhP as filler (CaPhP_a_5) and its blends with CHS-EPOX 520 (CaPhP_epox), curing agents Jeffamines (CaPhP_Jeff) and with dispersing agent BYK 9076 (CaPhP_BYK). The gray lines show the diffraction patterns with pronounced intensity of the peaks other than the (001) peak.

Such good compatibility could be attributed to the hybrid character of the particles when the phenyl groups (as organic moieties) enhance interactions with the polymer chains in the polymer blend. As can be seen from the powder XRD patterns of the unexfoliated CaPhP particles in the free cured epoxy film (see Figure 8, CaPhP_a_5), the main reflection is shifted to smaller angles, which indicates an enlargement of the basal spacing up to 15.6 Å. To determine which component of the film influences the basal spacing of CaPhP, the X-ray diffraction patterns of CaPhP with amino groups containing curing and dispersing agents used in the preparation of the films were also measured. The amines used as curing agents (Jeffamines) enlarge the basal spacing from 15.05 Å to 15.45 Å (CaPhP_Jeff). The reaction of CaPhP with BYK 9076, which is used as a dispersing agent in the preparation of the composite film and is an alkyl ammonium salt of a high molecular weight copolymer, increases the basal spacing of CaPhP by 0.3 Å (CaPhP_BYK). On the contrary, the macromolecules of the epoxy resin alone do not cause enlargement of the basal spacing (CaPhP_epox). The values of the basal spacing are summarized in Table 1. The observed changes of the basal spacing in CaPhP_Jeff and CaPhP_BYK are small and most probably cannot be explained by penetration of the long-chain macromolecules of Jeffamines or BYK 9076 into the structure, as it was observed for short alkylamines in the previous studies [19–20]. More likely the macromolecules surround the particles and the present amine groups interact with the phenyl groups on the edge of the particles and prevent them from free movement. This leads to a slight rearrangement inside the layers and thus to a small increase of the basal spacing. The presence of the amino groups seems to be important because no change of the basal spacing was observed for the epoxy resin alone.

**Table 1:** Comparison of the basal spacings for calcium phenylphosphonate alone, as a filler in the free film and its mixtures with individual components of the polymer blend.

sample	basal spacing, Å

CaPhP_a	15.02
CaPhP_epox	15.05
CaPhP_Jeff	15.45
CaPhP_BYK	15.32
CaPhP_a_5	15.61

The filler particles in the films are highly preferably oriented, as follows from the much lower intensity of the main peak in the diffraction pattern measured in the transmission mode compared to that measured in the reflection mode. In the case of the exfoliated particles only a smaller enlargement was observed, if at all (for the XRD patterns of the exfoliated particles see Supporting Information File 1). As it was mentioned previously, the monolamellar particles form only a part of the filler after the exfoliation treatment, thus there are also incompletely exfoliated particles, containing up to 20 layers.

#### Dynamic mechanical properties

**Figure 9:**
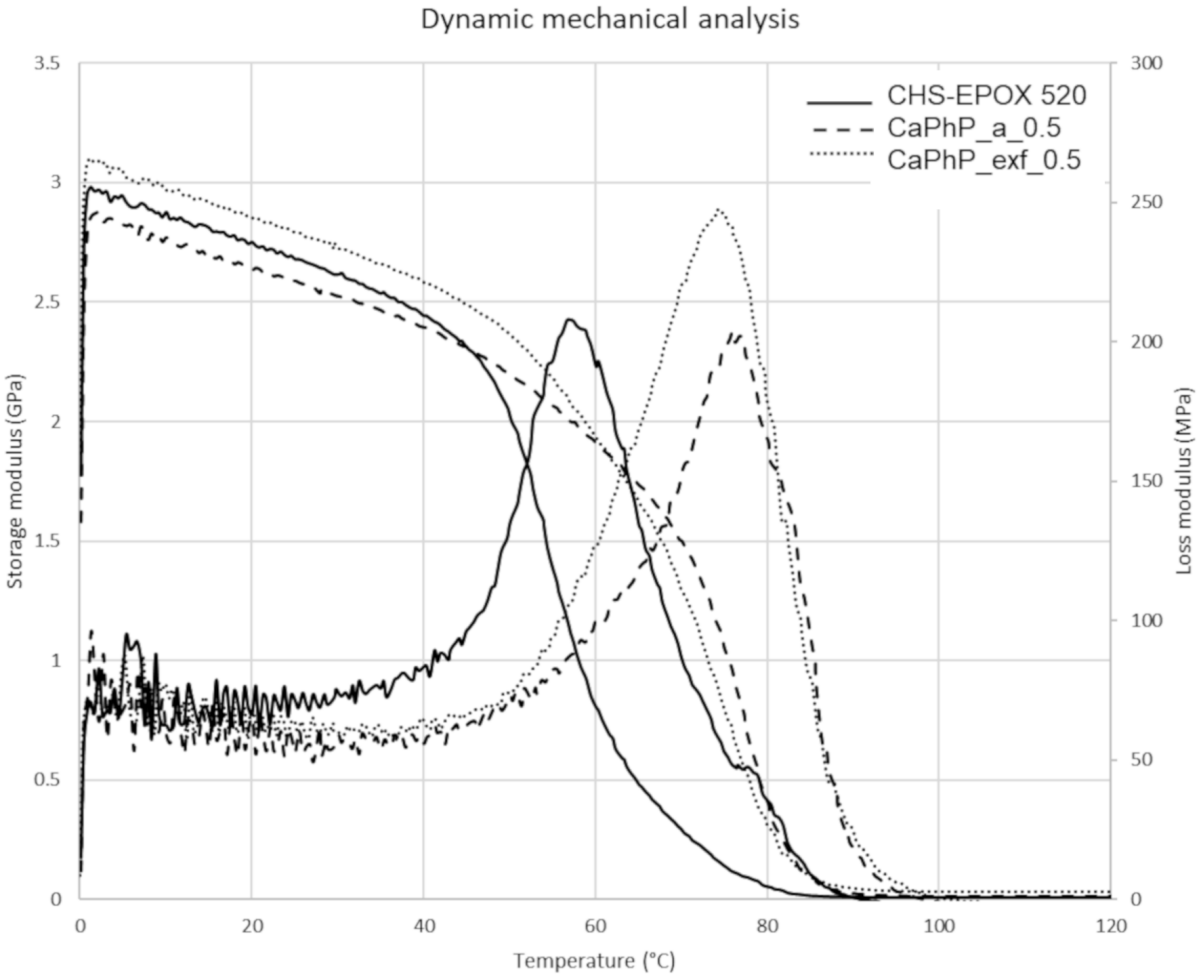
Storage and loss moduli measured by dynamic mechanical analysis for the pristine epoxy-free film (CHS-EPOX 520) and for free films loaded with 0.5 wt % of unexfoliated (CaPhP_a_0.5) and exfoliated (CaPhP_exf_0.5) filler.

The effect of the exfoliation on the mechanical properties was studied by comparing the free films containing the same amount of the filler (0.5 wt %), both unexfoliated (CaPhP_a_0.5) and exfoliated (CaPhP_exf_0.5). In the temperature range from 0 °C to 45 °C the exfoliated particles increase the storage modulus compared to that of the pristine epoxy matrix, while the unexfoliated filler decreases it (see Figure 9). For the 0.5 wt % load, the glass transition temperature of the composite films is shifted to higher values compared to the pristine film. While the value of the loss modulus of the composite with unexfoliated CaPhP is roughly the same as for the pristine epoxy film, the loss modulus of the composite with exfoliated CaPhP is higher.

#### Barrier properties

The addition of thin but large particles can influence the barrier properties of polymer films. In the case of CaPhP, the permeability of the epoxy film with 5% CaPhP (CaPhP_ a_5) as a filler for H_2_, CO_2_, He and CH_4_ was measured and the results were 21.02, 4.28, 13.55 and 4.85 Barrer, respectively (Table 2). This implies that the highest permeability was for H_2_ and the smallest for CO_2_. The addition of the filler increased the permeability for both these gases. In the case of hydrogen, it was approximately four times higher and in the case of CO_2_ even 5.6 times higher in comparison to the pristine epoxy film.

**Table 2:** Results of gas permeability measurements. The permeability of pristine epoxy film for He and CH_4_ were not measured.

	permeability (Barrer)			

sample	H_2_	CO_2_	He	CH_4_

CaPhP_a_5	21.02	4.28	13.55	4.85
pristine epoxy film	5.01	0.76	–	–

#### Flammability

Phosphorus containing compounds are used as flame retardant agents as an alternative to halogenated compound, which are effective but, on the other hand, they are considered to be hazardous for the environment and human health [21]. The structure of CaPhP contains a high amount of phosphorus as well as bound water molecules and seemed promising for this kind of application. A composite containing 0.5 wt % of the filler was tested according to ISO 4589-2 and the limiting oxygen index was determined (LOI – the percentage of oxygen in atmosphere that the polymer specimen needs to burn). In the case of exfoliated particles, the system showed no improvement , whereas in the case of unexfoliated particles, there was a small increase in the LOI. Therefore, the system with 5 wt % of unexfoliated particles was tested. This amount of filler increased the LOI from 19 to 21. As 5% of the unexfoliated filler is usually the maximal content in order to maintain the mechanical properties of the polymer, it will not meet the criteria for the flame retardancy with CaPhP alone. However, even a small improvement could be useful in combination with other flame retardants. This feature will be further investigated.

## Conclusion

The aim of this work was to study layered calcium phenylphosphonate, CaC_6_H_5_PO_3_∙2H_2_O, as a potential filler for an epoxy resin. This material was chosen as it can be incorporated into a polymer matrix not only as a bulk material but it can also be used as a precursor for the preparation of nanosheets by exfoliation and can thereby serve as a component of nanocomposites. The synthesis procedure was revised and the reaction conditions, under which this material can be prepared in a form suitable for exfoliation in a sufficient amount, were found. Furthermore, the conditions for the exfoliation of this compound were studied. The most suitable method for exfoliation was determined to be a treatment in alcohols, namely propan-2-ol, by an action of strong shear force generated by high-shear dispersers. It was found that this material is compatible with the epoxy resin used. The particle distribution in the polymer matrix was homogenous and CaPhP does not form agglomerates either in an unexfoliated or exfoliated form. It follows from the comparison of the composites with the same filler loading that the exfoliated particles have a higher impact on the composite properties, as determined by dynamic mechanical analysis. This is in agreement with the generally accepted idea that a higher surface-to-volume ratio leads to an enhanced interaction of nanoparticles with the surrounding polymer matrix. To conclude, this organic–inorganic hybrid material on its own is compatible with an epoxy resin and does not require any special treatment to be dispersed well in a polymer matrix. In the case of the exfoliated particles, the dispersion is even better. Thus, layered calcium phenylphosphonate can be considered as a promising nanofiller for polymer composites.

## Experimental

### Materials and methods

Phenylphosphonic acid (PhP), calcium chloride (CaCl_2_), ammonia solution, propan-2-ol (all Sigma-Aldrich); CHS-EPOXY 520 (Spolchemie, a. s., Czech Republic) – a low molecular weight epoxy resin based on bisphenol A; Jeffamine D230 and Jeffamine D2000 (Huntsman International LLC) – polyether amines were used. BYK 9076 – an alkylammonium salt of a high molecular weight copolymer and BYK 066 and a solution of foam-destroying polysiloxanes (both BYK-CHEMIE GMBH, Germany) were also used. All chemicals were used as obtained.

The characterization of the samples was performed using the following techniques and devices. The topological profile of the particles was measured by AFM with a Dimension ICON instrument, Bruker, Germany, in peak force mode with a ScanAsyst tip. The dynamic mechanical properties were measured with a Discovery hybrid rheometer, DHR2, TA Instruments. The experiment was performed in tension mode with a deformation of 0.1% and frequency of 1 Hz. The heating rate was set to 3 °C/min. Pictures of the free films were obtained from an Olympus BX51 optical microscope equipped with a DP70 digital camera system in addition to a JEOL SEM JSM-55000 LV with an EDX detector (GRESHAM Sirius 10, JEOL, USA Inc.) with an acceleration voltage of 20 kV. Powder X-ray diffraction data were obtained with a D8-Advance diffractometer, Bruker, Germany, with a Bragg–Brentano θ–θ geometry and with an EMPYREAN diffractometer, PANalytical, Netherlands (in both cases using Cu Kα radiation). The barrier properties for gas permeation were measured at 25 °C for free film samples of area 2 cm^2^. The limiting oxygen index was evaluated according to ISO 4589-2.

### Synthesis of calcium phenylphosphonate (CaPhP)

First, phenylphosphonic acid (7.9 g, 5 × 10^−2^ mol) was dissolved in 100 mL and a pH of the obtained solution was adjusted to 9 by adding concentrated aqueous ammonia solution. Then, 50 mL of CaCl_2_ solution (5.5 g, 5 × 10^−2^ mol) was added: a) at once (sample denoted as CaPhP_a); b) "drop by drop" (CaPhP_d); and c) in several portions (CaPhP_s). A white precipitate was formed immediately in all cases. Then, the reaction mixture was diluted by 50 mL of distilled water and stirred at medium speed (≈250 rpm) for 30 minutes. The precipitate formed was collected by filtration, washed with water until neutral pH to remove the remaining ammonia, and dried at room temperature. To obtain a fine powder it was possible to grind dried material in a friction bowl.

### Exfoliation of CaPhP

#### Solvent selection

A sample of CaPhP_a (10–13 mg) was put into a small glass vial to which 5 mL of a solvent (distilled water, propan-2-ol, ethanol, butanol, acetone) was added. The mixture was sonicated in an ultrasound bath (*f* = 37 kHz) for one hour and the temperature of the bath was cooled by adding ice. The quality and stability of the resulting dispersion was visually observed and photographs were taken immediately, 1 h, and 24 h after the ultrasound treatment. The presence of nanoparticles in dispersion after 24 h was confirmed by Tyndall scattering using a green laser (λ = 532 nm).

#### Preparation of stock dispersion of exfoliated particles

CaPhP_a (1.5 g, dry fine powder) was dispersed in 300 mL of propan-2-ol (*c* = 5 g/L). This dispersion was treated using an IKA T10 Standard Ultra-turrax^®^ (a high-shear force disperser, IKA^®^-Werke GmbH & Co. KG, Germany) equipped with a dispergation tool (S 10 D-7 G-KS-65) for 5 minutes at 13,000 rpm. A mixture of fully and partly exfoliated particles was obtained and denoted as CaPhP_exf.

### Preparation of polymer blends

#### Unexfoliated particles

First, 90 g of CHS-EPOXY 520 was heated to 80 °C and the dispersant BYK 9076 (9.5 g) was added and mixed with the polymer matrix using a dispersing disc. Then a fine powder of CaPhP_a (10 g) was added in small portions. The whole blend was mixed at 350 rpm 4 hours. After cooling down, the mixture was processed by a three-roll mill ten times with a 5 µm width of the slot between the rotating cylinders and rotation speed of 200 rpm. This polymer paste was used as a stock dispersion; from that, 10 g samples with a filler concentration of 0.5, 1, 3 and 5 wt % (denoted as CaPhP_a_0.5 to CaPhP_a_5, where the number indicates the amount of the filler added) were prepared by diluting with pristine CHS-EPOXY 520. Finally, a BYK 066 defoaming agent (0.05 g) was added under careful stirring with a glass stick into each sample.

#### Exfoliated particles

The dispersion of CaPhP_exf in an amount corresponding to 0.05, 0.1, 0.3 and 0.5 wt % was added to CHS-EPOXY 520 (10 g). The mixture was stirred with a dispersive disc and heated to 80 °C until most of the propan-2-ol was evaporated. Then the BYK 066 defoaming agent (0.05 g) was added into each sample under stirring with a glass stick. The samples were denoted as CaPhP_exf_0.05 to CaPhP_exf_0.5, where the number indicates the weight percentage of CaPhP_exf in the final product.

### Preparation of free films

Prepared dispersions with the exfoliated and unexfoliated fillers were thoroughly mixed by hand with a mixture of curing agents (3.1 g of Jeffamine D230 and 0.78 g of Jeffamine D2000) and defoamed in a desiccator for a few minutes. Then the free films were prepared on polypropylene plates using a 150 µm gap applicator. The films were cured for one day at a room temperature and then for 1 h at 40 °C, 4 hours at 60 °C and finally 20 h at 80 °C. The cured films were collected from the supporting plates with the help of a razor blade.

## Supporting Information

File 1 XRD patterns of exfoliated sample CaPhP_exf and XRD pattern of free film with exfoliated filler CaPhP_exf_0.5.
The XRD patterns of exfoliated sample CaPhP_exf prepared by spin coating on the glass support (A) and XRD pattern of free film with exfoliated filler CaPhP_exf_0.5 (B).
