# Supplementary figures and images for: Layered calcium phenylphosphonate: a hybrid material for a new generation of nanofillers
Source: Beilstein J Nanotechnol. 2018 Nov 20;9:2906–15. doi: 10.3762/bjnano.9.269 (PMC6278773; doi:10.3762/bjnano.9.269)

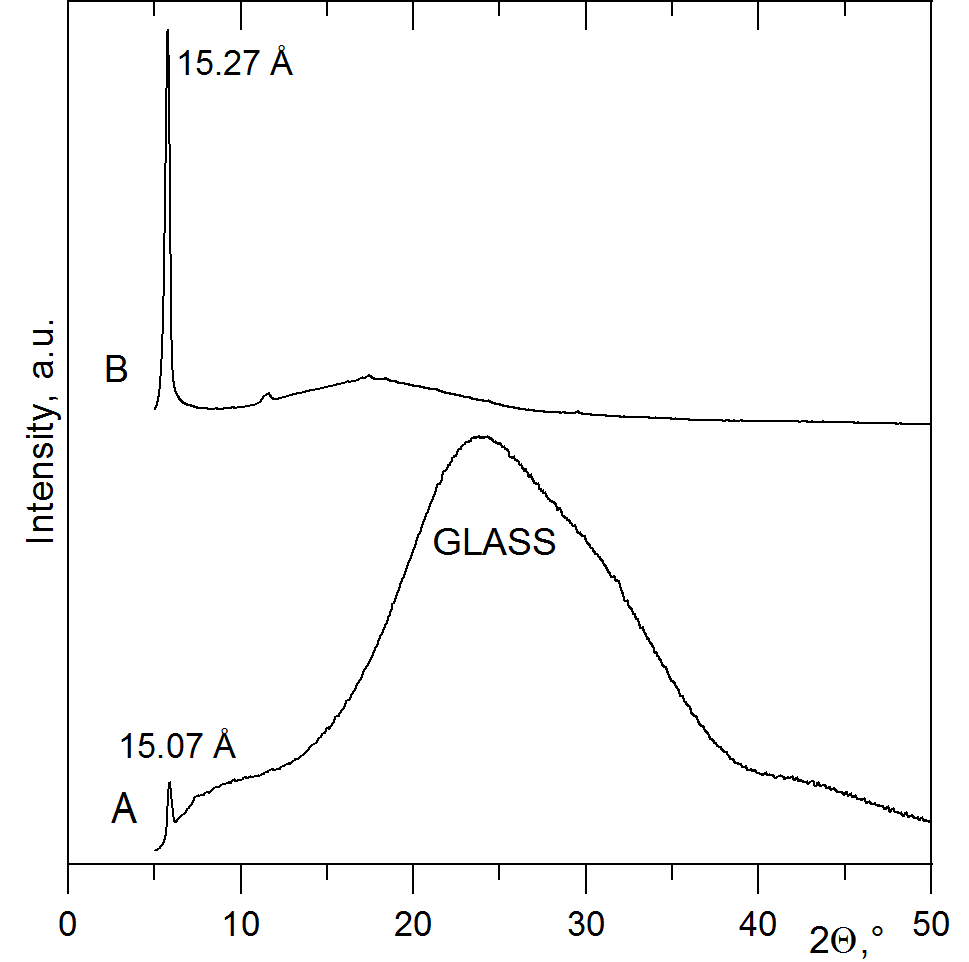

Supplement: File 1 — XRD patterns of exfoliated sample CaPhP_exf and XRD pattern of free film with exfoliated filler CaPhP_exf_0.5. The XRD patterns of exfoliated sample CaPhP_exf prepared by spin coating on the glass support (A) and XRD pattern of free film with exfoliated filler CaPhP_exf_0.5 (B). [file Beilstein_J_Nanotechnol-09-2906-s001.png]
